# AK112 (PD-1/VEGF-a bispecific antibody) combined with chemotherapy in locally advanced pancreatic cancer: a case report

**DOI:** 10.3389/fonc.2025.1667445

**Published:** 2026-01-09

**Authors:** Jiuliang Yan, Hanguang Dong, Chuntao Wu, Haitao Gu, Zihao Qi, Tao Chen, Beiyuan Hu, Huijiang Zhou, Jiang Long

**Affiliations:** 1 Department of Pancreatic Surgery, Shanghai General Hospital, Shanghai Jiao Tong University School of Medicine, Shanghai, China; 2 Shanghai Key Laboratory of Pancreatic Disease, Institute of Pancreatic Disease, Shanghai Jiao Tong University School of Medicine, Shanghai, China

**Keywords:** AK112, bispecific antibody, chemotherapy, locally advanced pancreatic cancer, PD-1, VEGF-A

## Abstract

**Background:**

Pancreatic ductal adenocarcinoma (PDAC) is one of the most malignant tumors. Most patients are diagnosed at advanced or metastatic stages, losing the opportunity for curative surgery. Current systemic treatments for pancreatic cancer remain suboptimal.

**Case summary:**

A 68-year-old female presented with upper abdominal pain. Imaging revealed a pancreatic mass, prompting an exploratory laparotomy. Intraoperative frozen section pathology identified metastatic adenocarcinoma nodules near the hepatic artery. The patient was subsequently evaluated and enrolled in a clinical trial. The treatment regimen comprised AG chemotherapy (nab-paclitaxel and gemcitabine) combined with AK112 (a novel bispecific antibody). After 13 administrations of AG chemotherapy and 9 infusions of AK112, imaging evaluation demonstrated partial tumor regression. A multidisciplinary team (MDT) assessment deemed the lesion potentially resec]. The exploratory laparotomy confirmed resectability, and a total pancreatectomy was performed. Postoperative pathology confirmed moderately differentiated pancreatic ductal adenocarcinoma with focal degenerative changes. During postoperative treatment, wound exudate suggestive of intestinal leakage prompted surgical intervention on June 12, 2023. Adjuvant therapy resumed after wound healing but was discontinued in September 2023 due to recurrent incision leakage. Maintenance therapy with tegafur was initiated thereafter. Unfortunately, the patient died of acute myocardial infarction on August 4, 2024.

**Conclusion:**

The combination therapy of AK112 and AG achieved good results in this case, but the broader efficacy of this regimen across the pancreatic cancer population awaits validation through large-scale clinical trials. The ongoing trials are highly anticipated, as successful results could establish AK112 as a novel therapeutic strategy for pancreatic cancer.

## Introduction

Pancreatic ductal adenocarcinoma (PDAC) is one of the most malignant tumors. Due to its insidious onset, challenges in early diagnosis, and rapid progression, most patients are diagnosed at advanced or metastatic stages, losing the opportunity for curative surgery. Furthermore, PDAC exhibits resistance to conventional anti-tumor therapies, resulting in a poor prognosis. Current systemic treatments for pancreatic cancer remain suboptimal. This report presents a case of locally advanced pancreatic cancer that achieved favorable therapeutic outcomes through a novel bispecific antibody, AK112, combined with chemotherapy. AK112 is a human immunoglobulin G1 (IgG1) monoclonal antibody (mAb) engineered as a bispecific molecule targeting both programmed cell death-1 (PD-1) and vascular endothelial growth factor-A (VEGF-A). Structurally, the anti-PD-1 single-chain variable fragment (ScFv) is conjugated to the C-terminus of each heavy chain of the anti-VEGF-A antibody. This design enables AK112 to simultaneously bind VEGF-A, a key mediator of tumor angiogenesis, and PD-1, an inhibitory receptor predominantly expressed on activated T cells. This case demonstrates that AK112 combined chemotherapy may be a new treatment strategy for locally advanced pancreatic cancer.

## Case presentation

A 68-year-old female presented to a local hospital with upper abdominal pain in January 2022. Imaging studies revealed an approximately 4.4 cm × 2.1 cm mass in the neck and body of the pancreas. The serum CA19–9 level was elevated at 36 U/mL (0-25 U/mL). Consequently, an exploratory laparotomy was performed at the local hospital. Intraoperative frozen section pathology identified metastatic adenocarcinoma nodules near the hepatic artery. As complete tumor resection was unfeasible, the procedure was aborted. The patient was subsequently referred to our hospital in March 2022 for evaluation and enrollment in a clinical trial. The treatment regimen comprised AG chemotherapy (nab-paclitaxel: 125 mg/m², gemcitabine: 1 g/m² on days 1, 8, and 15 of a 28-day cycle) combined with AK112 (734 mg on day 1 every 2 weeks). Dose adjustments were permitted for severe adverse events.

**Figure 1:**
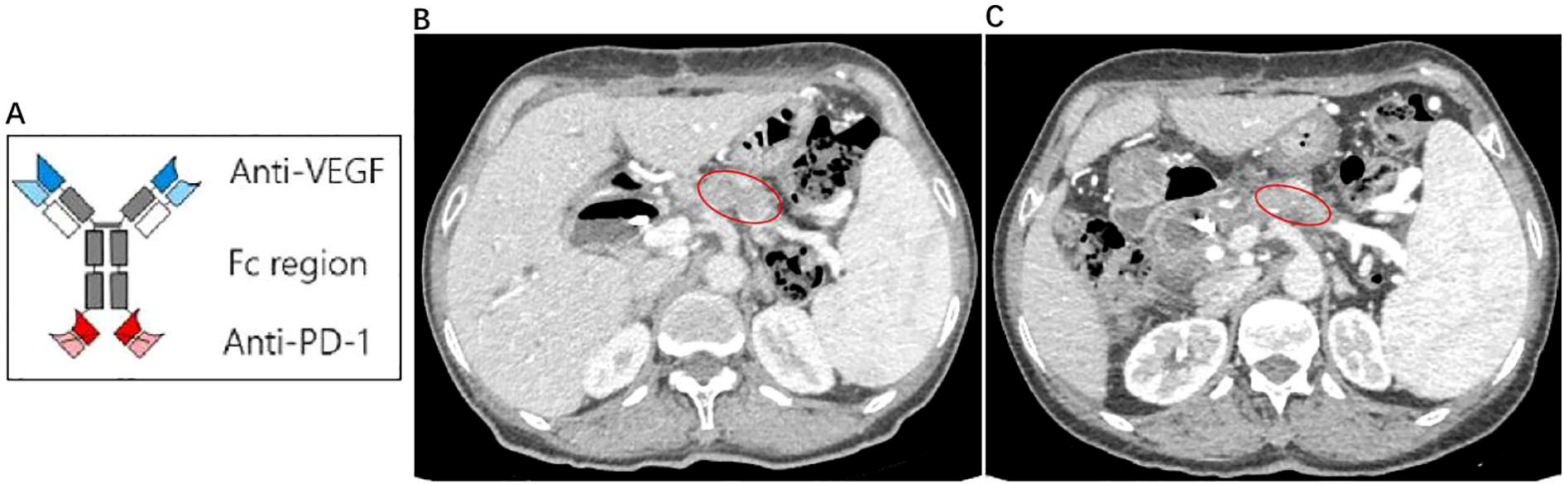
**(A)** Structural pattern diagram of AK112:AK112 is a human immunoglobulin G1 monoclonal antibody, which is a bispecific antibody anti-PD-1/VEGF-A. Its anti-PD-1 ScFv is attached to the C-terminus of each anti-VEGF-A antibody heavy chain; **(B)** The pretreatment CT scan reveals a tumor measuring approximately 44.19mm × 21.34mm; **(C)** The posttreatment CT scan demonstrates a tumor measuring approximately 35.49mm × 16.11mm.

**Figure 2:**
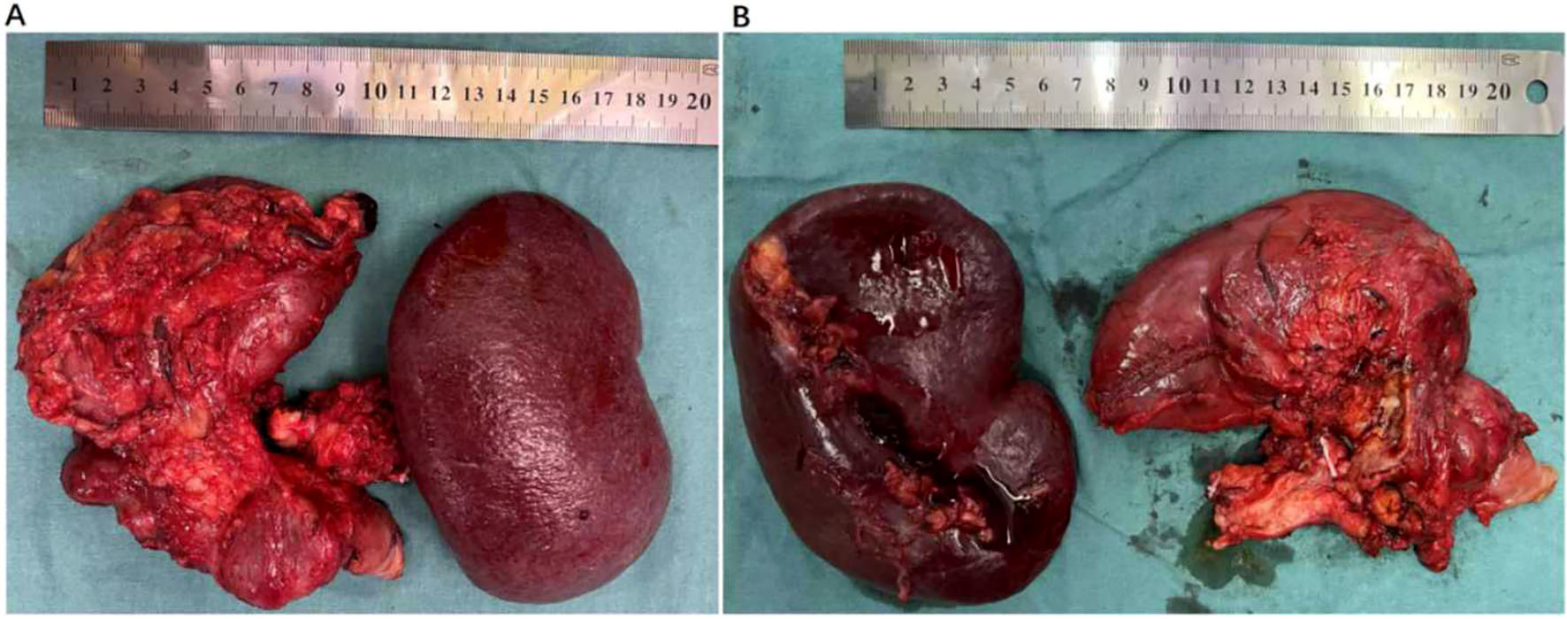
Postoperative specimen following total pancreatectomy. **(A)** Anterior view. **(B)** Posterior view.

Following the first cycle, the patient developed bone marrow suppression (nab-paclitaxel 162.5mg, gemcitabine 1.3g), prompting dose reductions to nab-paclitaxel 125mg and gemcitabine 1.0g. Recurrent bone marrow suppression during the fifth cycle necessitated further reductions to nab-paclitaxel 97.5mg and gemcitabine 0.78g. According to RECIST 1.1 criteria, tumor size decreased by approximately 20% following 13 administrations of AG chemotherapy and 9 infusions of AK112 (**Figure 1**). A multidisciplinary team (MDT) assessment deemed the lesion potentially resectable. On December 12, 2022, exploratory laparotomy confirmed resectability, and a total pancreatectomy was performed (**Figure 2**). Postoperative histopathological examination confirmed a moderately differentiated pancreatic ductal adenocarcinoma with focal degenerative changes. The tumor was found to infiltrate the duodenal wall, extending to the mucosal layer, and demonstrated perineural invasion into local small nerves, with no definite evidence of vascular invasion. Surgical margins were negative. Lymph node examination revealed no metastatic involvement in the surrounding lymph nodes; however, metastatic cancer nodules were identified in the stomach—one along the lesser curvature and three along the greater curvature. Genetic analysis identified somatic mutations in KRAS p.G12C and TP53 p.C238Y, with no germline mutations detected. The tumor was microsatellite stable (MSS).

**Figure 3:**
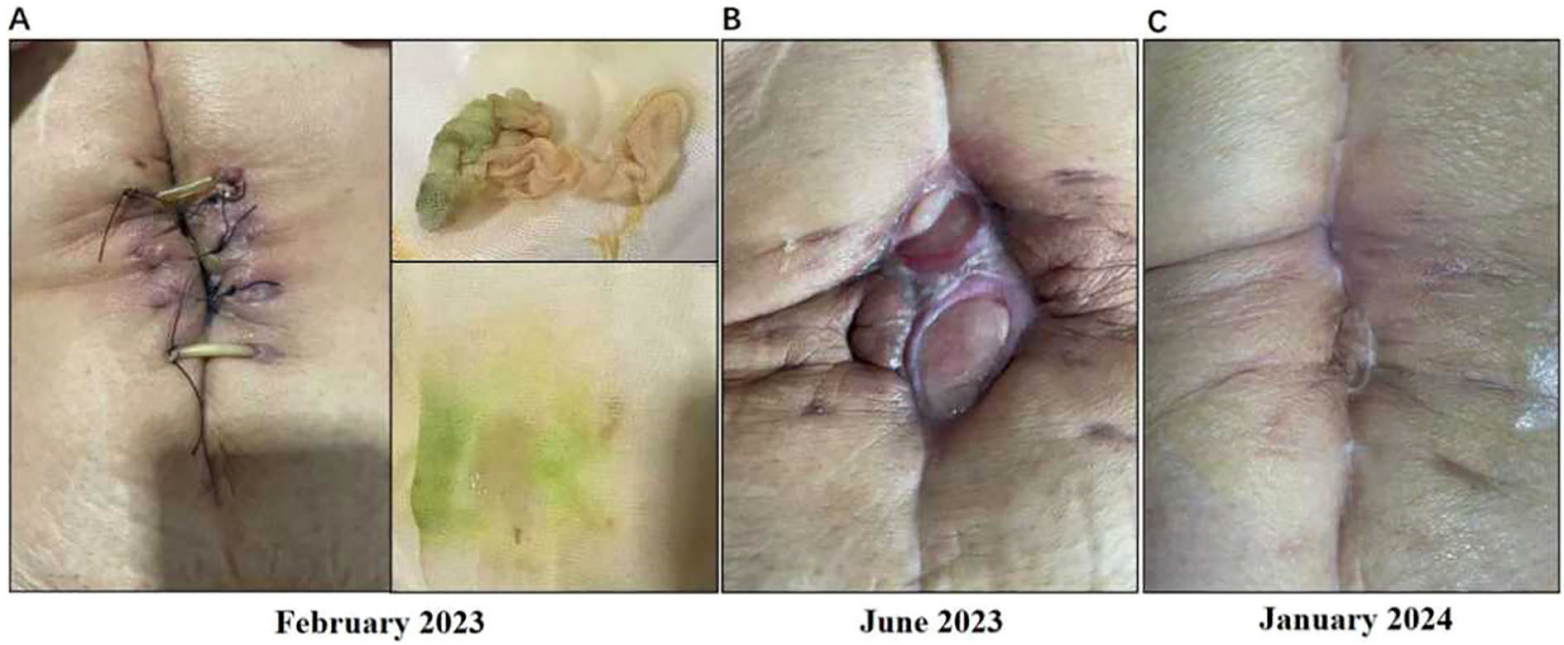
**(A)** The patient experienced delayed wound healing and significant exudation after the first surgery; **(B)** During postoperative adjuvant therapy, the patient experienced wound exudation again; **(C)** After stopping postoperative adjuvant therapy, the patient’s wound completely healed.

**Figure 4:**
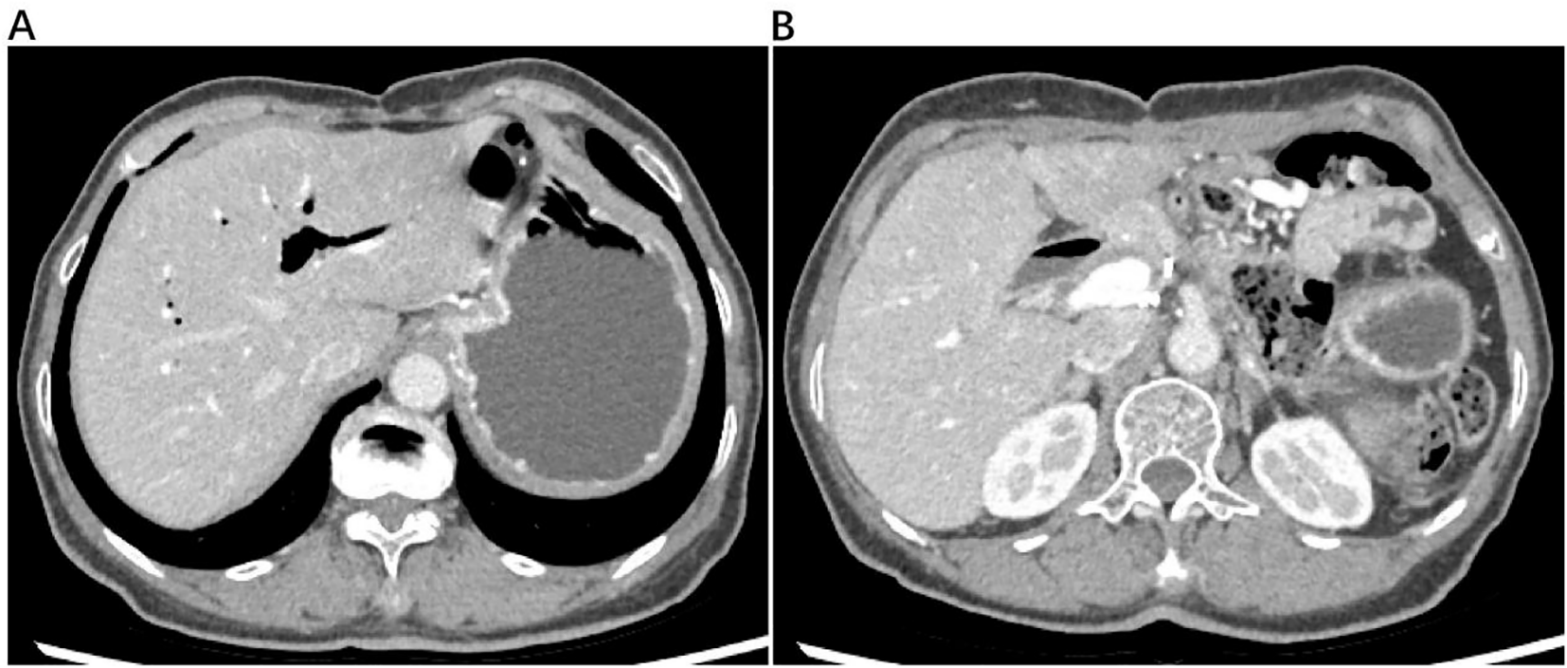
An abdominal CT scan, obtained for routine follow-up on June 12, 2024, demonstrated partial dilation of the intrahepatic bile ducts with pneumobilia **(A)**. The CT scan revealed no radiological evidence of tumor recurrence **(B)**.

Due to delayed wound healing, postoperative adjuvant therapy (AG + AK112) was delayed until April 18, 2023. During postoperative treatment (4 administrations of AG and 2 infusions of AK112), wound exudate suggestive of intestinal leakage prompted surgical intervention on June 12, 2023 (small bowel perforation repair and abdominal debridement) (**Figure 3**). Adjuvant therapy resumed after wound healing but was discontinued in September 2023 due to recurrent incision leakage. Maintenance therapy with tegafur was initiated thereafter. A routine follow-up abdominal CT scan on June 12, 2024, revealed partial intrahepatic bile duct dilation with pneumobilia, without evidence of tumor recurrence (**Figure 4**).

**Figure 5:**
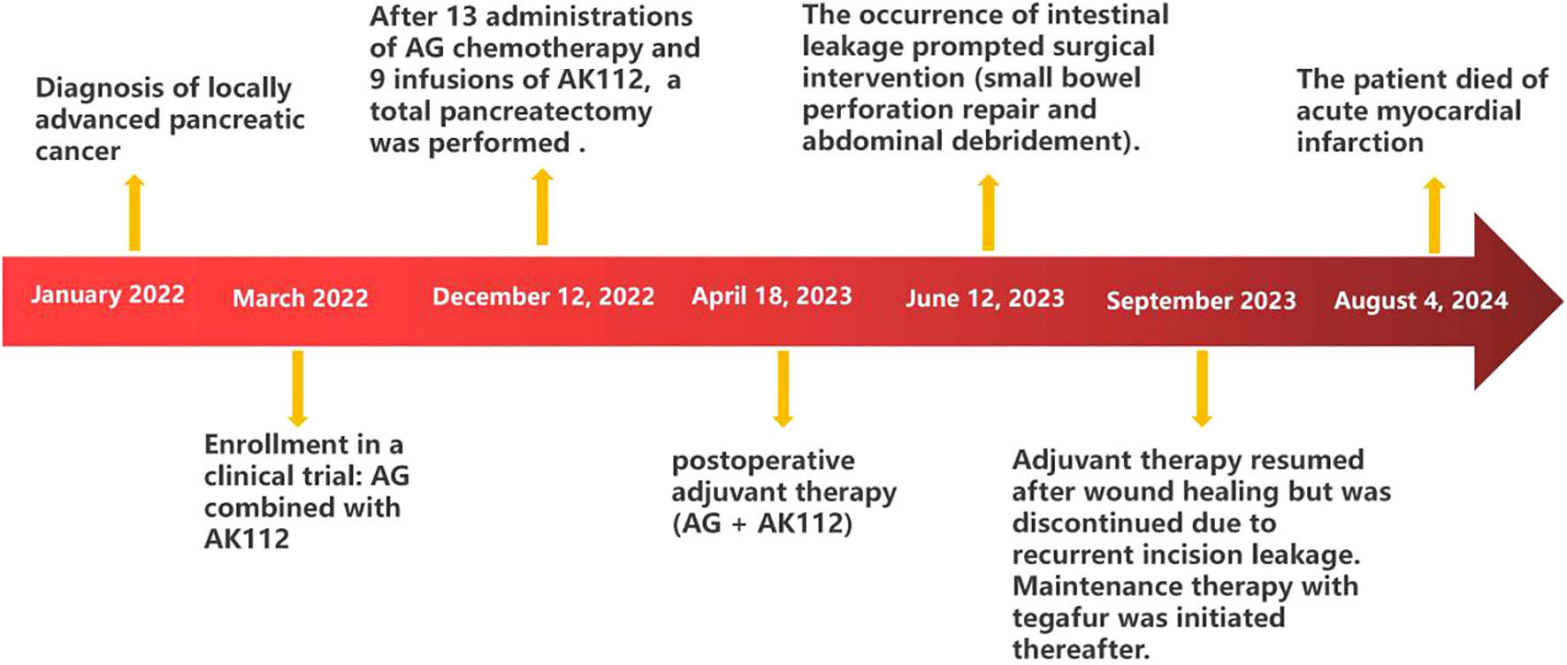
The timeline diagram outlines the patient’s clinical course from initial diagnosis through treatment to death.

At approximately 02:00 a.m. on August 4, 2024, the patient was urgently admitted to a local hospital following the acute onset of nausea, vomiting, and back pain. Subsequent diagnostic evaluation revealed a markedly elevated serum troponin I level of >25.61 ng/mL, leading to a provisional diagnosis of acute myocardial infarction. Despite the immediate implementation of aggressive resuscitative measures, the patient deteriorated and was pronounced deceased at 09:38 on the same day. The timeline diagram outlines the patient’s clinical course, from initial diagnosis through treatment to death (**Figure 5**).

## Discussion

Pancreatic cancer remains a highly lethal malignancy with a 5-year survival rate below 12% (1). Its genetic complexity and immunosuppressive tumor microenvironment (TME) contribute to resistance to radiotherapy, chemotherapy, targeted therapy, and immunotherapy. For advanced PDAC, gemcitabine-based regimens have been the cornerstone of systemic therapy. In 1997, gemcitabine was approved as the first-line treatment for pancreatic cancer following a randomized trial demonstrating its superiority over 5-fluorouracil (5-FU). The trial reported a longer median survival time (5.65 vs. 4.41 months) and a higher 12-month survival rate (18% vs. 2%) in advanced PDAC patients (2). Subsequent research explored gemcitabine in combination with other agents or compared it to alternative chemotherapy regimens, though most studies failed to meet their primary endpoints. A pivotal shift occurred in 2011 when a clinical trial by Conroy et al. demonstrated that FOLFIRINOX (a combination of oxaliplatin, irinotecan, fluorouracil, and leucovorin) provided a significant survival advantage over gemcitabine monotherapy. The study reported a median overall survival (OS) of 11.1 months versus 6.8 months and a median progression-free survival (PFS) of 6.4 months versus 3.3 months, albeit with increased toxicity (3). This established FOLFIRINOX as a viable option for patients with advanced or metastatic pancreatic cancer in good clinical condition. In 2013, Von Hoff et al. further showed that the combination of albumin-bound paclitaxel and gemcitabine (AG) improved outcomes compared to gemcitabine alone, with median OS of 8.5 vs. 6.7 months, 1-year survival rates of 35% vs. 22%, and 2-year survival rates of 9% vs. 4%. The AG group also exhibited a longer median PFS (5.5 vs. 3.7 months), though toxicity remains a limiting factor for novel agent combinations (4).

Angiogenesis has emerged as a potential therapeutic target. Pysz et al. observed enhanced ultrasound signals of vascular endothelial growth factor receptor type 2 (VEGFR-2) in pancreatic tumors compared to normal tissue in mouse models, particularly in tumors smaller than 3 mm (5). While this suggests angiogenesis markers could aid early detection, clinical trials of VEGF inhibitors such as sorafenib—alone or combined with gemcitabine—failed to improve outcomes in advanced pancreatic cancer (6). KRAS mutations, present in over 90% of pancreatic cancers, remain a critical focus. Sotorasib and adagrasib effectively target the KRAS G12C mutation, but this variant represents only 2–3% of cases. The more prevalent mutations—G12D (44%), G12V (34%), and G12R (20%)—highlight the need for broader inhibitors. MRTX1133, a KRAS G12D inhibitor, is currently under clinical investigation (7). In KRAS wild-type patients, nimotuzumab combined with gemcitabine significantly improved median OS (8.6 vs. 6.0 months; P = 0.0341) and PFS (5.1 vs. 3.4 months; P = 0.0163) (8). Additionally, the POLO trial demonstrated that maintenance olaparib prolonged PFS in patients with germline BRCA mutations (9).

Although immunotherapy has demonstrated progress in treating various solid tumors, its clinical efficacy in pancreatic cancer remains limited, which only benefits less than 1% of patients (10). Clinical trials of immune checkpoint inhibitors in locally advanced pancreatic cancer have yielded inconsistent results. While some studies report modest improvements in PFS, the majority have demonstrated limited success, with a median PFS ranging from 4 to 5 months (11–15). Two primary factors contribute to this resistance: 1) the immune escape mechanisms inherent to pancreatic cancer, and 2) its low tumor mutational burden combined with a highly fibrotic and hypoxic TME, which fosters immunosuppression. Pembrolizumab is currently the only FDA-approved second-line therapy for microsatellite instability-high (MSI-H) or mismatch repair-deficient (dMMR) solid tumors, including pancreatic cancer. However, clinical data reveal modest outcomes, with an objective response rate (ORR) of 18% and a median PFS of 2.2 months in pancreatic cancer patients (16). Similarly, a phase Ib/II trial evaluating durvalumab combined with ibrutinib in relapsed/refractory solid tumors reported an ORR of 2%, a median OS of 4.2 months, and a median PFS of 1.7 months in pancreatic cancer cohorts (17). Another phase II randomized trial in metastatic pancreatic cancer showed an ORR of 3.1% for durvalumab plus tremelimumab, while durvalumab monotherapy elicited no response (18).

Emerging evidence suggests that cytotoxic agents and local ablation therapies may enhance immunotherapy efficacy by inducing immunogenic cell death, disrupting immune evasion, and alleviating immunosuppression within the TME (19). Furthermore, preclinical studies highlight synergistic effects between immunotherapy and targeted therapies, such as anti-angiogenic agents and tyrosine kinase inhibitors (20). These findings underscore the need for innovative combinatorial approaches. Future strategies should integrate novel immunotherapies with cytotoxic drugs, localized ablation, or targeted therapies to counteract tumor-driven immune escape mechanisms and improve clinical outcomes.

Beyond its role in promoting angiogenesis, VEGF-A exerts immunosuppressive effects within the TME. High concentrations of VEGF in the TME not only suppress the effector capabilities of cytotoxic T lymphocytes and impede the maturation and antigen presentation of dendritic cells, but also stimulate the recruitment and expansion of immunosuppressive cells, such as Tregs, myeloid-derived suppressor cell, and tumor-promoting M2-like macrophages. VEGF promotes the development of defective tumor blood vessels that exhibit both structural and functional irregularities, leading to an abnormal TME under aberrant conditions of interstitial hypertension, hypoxia, and acidosis. These conditions further promote immunosuppressive effects at local and systemic levels (21–23). VEGF-A has been shown to upregulate PD-1 expression in tumor-infiltrating CD8+ T cells. Notably, evidence reveals a strong correlation between VEGF-A and PD-1 co-expression in the TME (24). Targeting the VEGF/VEGFR pathway in cancer exerts a dual impact, combining anti-angiogenesis with immune support. This activity may thereby shift the tumor phenotype from immunologically “cold” to “hot”. Preclinical studies have consistently revealed that the combination of VEGF/VEGFR and PD-1/PD-L1 inhibitors produces a robust synergistic effect. This therapeutic synergy has been demonstrated in diverse tumor types, such as glioblastomas, melanoma, lung cancer, and hepatocellular carcinoma (25, 26). The therapeutic potential of this strategy is being explored in clinical trials for a range of malignancies, including recurrent glioblastoma, renal cell carcinoma, colorectal cancer, and ovarian cancer, and may yield promising outcomes for these aggressive, treatment-refractory tumors (27, 28).

Unlike combination therapies using separate anti-PD-1 and anti-VEGF antibodies, AK112 integrates dual targeting into a single therapeutic agent. By concurrently blocking these two pathways, AK112 may enhance localized drug accumulation and synergistically counteract immunosuppressive mechanisms in the TME.

## Conclusion

Although the combination therapy of AK112 and AG achieved good results in this case, the broader efficacy of this regimen across the pancreatic cancer population awaits validation through large-scale clinical trials. The ongoing trials are highly anticipated, as successful results could establish AK112 as a novel therapeutic strategy for pancreatic cancer.

## Data Availability

The original contributions presented in the study are included in the article/Supplementary Material. Further inquiries can be directed to the corresponding author.

## References

[1] Siegel RL Miller KD Wagle NS Jemal A . Cancer statistics, 2023. CA Cancer J Clin. (2023) 73:17–48. doi: 10.3322/caac.21763, PMID: 36633525

[2] Burris 3HA Moore MJ Andersen J Green MR Rothenberg ML Modiano MR . Improvements in survival and clinical beneft with gemcitabine as frst-line therapy for patients with advanced pancreas cancer: a randomized trial. J Clin Oncol. (1997) 15:2403–13. doi: 10.1200/JCO.1997.15.6.2403, PMID: 9196156

[3] Conroy T Desseigne F Ychou M Bouché O Guimbaud R Bécouarn Y . FOLFIRINOX versus gemcitabine for metastatic pancreatic cancer. N Engl J Med. (2011) 364:1817–25. doi: 10.1056/NEJMoa1011923, PMID: 21561347

[4] Von Hoff DD Ervin T Arena FP Chiorean EG Infante J Moore M . Increased survival in pancreatic cancer with nab-paclitaxel plus gemcitabine. N Engl J Med. (2013) 369:1691–703. doi: 10.1056/NEJMoa1304369, PMID: 24131140 PMC4631139

[5] Pysz MA Machtaler SB Seeley ES Lee JJ Brentnall TA Rosenberg J . Vascular endothelial growth factor receptor type 2-targeted contrast-enhanced US of pancreatic cancer neovasculature in a genetically engineered mouse model: potential for earlier detection. Radiology. (2015) 274:790–9. doi: 10.1148/radiol.14140568, PMID: 25322341 PMC4372059

[6] Gonçalves A Gilabert M François E Dahan L Perrier H Lamy R . BAYPAN study: a double-blind phase III randomized trial comparing gemcitabine plus sorafenib and gemcitabine plus placebo in patients with advanced pancreatic cancer. Ann Oncol. (2012) 23:2799–805. doi: 10.1093/annonc/mds135, PMID: 22771827

[7] Stickler S Rath B Hamilton G . Targeting KRAS in pancreatic cancer. Oncol Res. (2024) 32:799–805. doi: 10.32604/or.2024.045356, PMID: 38686056 PMC11055996

[8] Schultheis B Reuter D Ebert MP Siveke J Kerkhoff A Berdel WE . Gemcitabine combined with the monoclonal antibody nimotuzumab is an active first-line regimen in KRAS wildtype patients with locally advanced or metastatic pancreatic cancer: a multicenter, randomized phase IIb study. Ann Oncol. (2017) 28:2429–35. doi: 10.1093/annonc/mdx343, PMID: 28961832

[9] Golan T Hammel P Reni M Cutsem EV Macarulla T Hall MJ . Maintenance olaparib for germline BRCA-mutated metastatic pancreatic cancer. N Engl J Med. (2019) 381:317–27. doi: 10.1056/NEJMoa1903387, PMID: 31157963 PMC6810605

[10] Giurini EF Ralph O Pappas SG Gupta KH . Looking beyond checkpoint inhibitor monotherapy: uncovering new frontiers for pancreatic cancer immunotherapy. Mol Cancer Ther. (2025) 24:18–32. doi: 10.1158/1535-7163.MCT-24-0311, PMID: 39311547 PMC11694065

[11] Zhou B Zhang SR Chen G Chen P . Developments and challenges in neoadjuvant therapy for locally advanced pancreatic cancer. World J Gastroenterol. (2023) 29:5094–103. doi: 10.3748/wjg.v29.i35.5094, PMID: 37744290 PMC10514760

[12] Brozos-Vázquez E Toledano-Fonseca M Costa-Fraga N García-Ortiz MV Díaz-Lagares Á Rodríguez-Ariza A . Pancreatic cancer biomarkers: A pathway to advance in personalized treatment selection. Cancer Treat Rev. (2024) 125:102719. doi: 10.1016/j.ctrv.2024.102719, PMID: 38490088

[13] Gupta N Yelamanchi R . Pancreatic adenocarcinoma: A review of recent paradigms and advances in epidemiology, clinical diagnosis and management. World J Gastroenterol. (2021) 27:3158–81. doi: 10.3748/wjg.v27.i23.3158, PMID: 34163104 PMC8218366

[14] Zhu S Cheng Q Zou M Li C Tang Y Xia L . Combining bulk and scRNA-seq to explore the molecular mechanisms governing the distinct efferocytosis activities of a macrophage subpopulation in PDAC. J Cell Mol Med. (2024) 28:e18266. doi: 10.1111/jcmm.18266, PMID: 38501838 PMC10949604

[15] Barakat R Sidira D Stavropoulos A Konsolas N Palantzas A Filippou D . The possible role of immunotherapy in locally advanced pancreatic cancer treatment. Acta Med Acad. (2025) 54:143–51. doi: 10.5644/ama2006-124.479, PMID: 41231056 PMC12739874

[16] Marabelle A Le DT Ascierto PA Di Giacomo AM De Jesus-Acosta A Delord JP . Efficacy of pembrolizumab in patients with noncolorectal high microsatellite instability/mismatch repair-deficient cancer: results from the phase II KEYNOTE-158 study. J Clin Oncol. (2020) 38:1–10. doi: 10.1200/JCO.19.02105, PMID: 31682550 PMC8184060

[17] Hong D Rasco D Veeder M Luke JJ Chandler J Balmanoukian A . A phase 1b/2 study of the Bruton tyrosine kinase inhibitor ibrutinib and the PD-L1 inhibitor durvalumab in patients with pretreated solid tumors. Oncology. (2019) 97:102–11. doi: 10.1159/000500571, PMID: 31230047

[18] O’Reilly EM Oh DY Dhani N Renouf DJ Lee MA Sun W . Durvalumab with or without tremelimumab for patients with metastatic pancreatic ductal adenocarcinoma: a phase 2 randomized clinical trial. JAMA Oncol. (2019) 5:1431–8. doi: 10.1001/jamaoncol.2019.1588, PMID: 31318392 PMC6647002

[19] Galluzzi L Humeau J Buqué A Zitvogel L Kroemer G . Immunostimulation with chemotherapy in the era of immune checkpoint inhibitors. Nat Rev Clin Oncol. (2020) 17:725–41. doi: 10.1038/s41571-020-0413-z, PMID: 32760014

[20] Zhou XH Hou WT Gao L Shui L Yi C Zhu H . Synergies of antiangiogenic therapy and immune checkpoint blockade in renal cell carcinoma: from theoretical background to clinical reality. Front Oncol. (2020) 10:1321. doi: 10.3389/fonc.2020.01321, PMID: 32850419 PMC7403214

[21] Gabrilovich DI Chen HL Girgis KR Cunningham HT Meny GM Nadaf S . Production of vascular endothelial growth factor by human tumors inhibits the functional maturation of dendritic cells. Nat Med. (1996) 2:1096–103. doi: 10.1038/nm1096-1096, PMID: 8837607

[22] Jain RK . Normalization of tumor vasculature: an emerging concept in antiangiogenic therapy. Science. (2005) 307:58–62. doi: 10.1126/science.1104819, PMID: 15637262

[23] Lyu L Yi M Chen J Zhang J Ma X Zhang X . Bispecific antibody targeting VEGF/TGF-β Synergizes with local radiotherapy: turning tumors from cold to inflamed and amplifying abscopal effects. Adv Sci (Weinh). (2025) 12:e01819. doi: 10.1002/advs.202501819, PMID: 40470716 PMC12376579

[24] Voron T Colussi O Marcheteau E Pernot S Nizard M Pointet AL . VEGF-A modulates expression of inhibitory checkpoints on CD8+ T cells in tumors. J Exp Med. (2015) 212:139–48. doi: 10.1084/jem.20140559, PMID: 25601652 PMC4322048

[25] Hodi FS Lawrence D Lezcano C Wu X Zhou J Sasada T . Bevacizumab plus ipilimumab in patients with metastatic melanoma. Cancer Immunol Res. (2014) 2:632–42. doi: 10.1158/2326-6066.CIR-14-0053, PMID: 24838938 PMC4306338

[26] Liu XD Hoang A Zhou L Kalra S Yetil A Sun M . Resistance to antiangiogenic therapy is associated with an immunosuppressive tumor microenvironment in metastatic renal cell carcinoma. Cancer Immunol Res. (2015) 3:1017–29. doi: 10.1158/2326-6066.CIR-14-0244, PMID: 26014097 PMC4561186

[27] Omuro A Vlahovic G Lim M Sahebjam S Baehring J Cloughesy T . Nivolumab with or without ipilimumab in patients with recurrent glioblastoma: results from exploratory phase I cohorts of CheckMate 143. Neuro Oncol. (2018) 20:674–86. doi: 10.1093/neuonc/nox208, PMID: 29106665 PMC5892140

[28] Tamura R Tanaka T Akasaki Y Murayama Y Yoshida K Sasaki H . The role of vascular endothelial growth factor in the hypoxic and immunosuppressive tumor microenvironment: perspectives for therapeutic implications. Med Oncol. (2019) 37:2. doi: 10.1007/s12032-019-1329-2, PMID: 31713115

